# Deficiency of the ER-stress-regulator MANF triggers progressive outer hair cell death and hearing loss

**DOI:** 10.1038/s41419-020-2286-6

**Published:** 2020-02-06

**Authors:** Anni Herranen, Kuu Ikäheimo, Tuuli Lankinen, Emmi Pakarinen, Bernd Fritzsch, Mart Saarma, Maria Lindahl, Ulla Pirvola

**Affiliations:** 1 Molecular and Integrative Biosciences Research Programme, University of Helsinki, 00014 Helsinki, Finland; 2 Institute of Biotechnology, HILIFE Unit, University of Helsinki, 00014 Helsinki, Finland; 3 Department of Biology, CLAS, University of Iowa, Iowa City, IA 52242-1324 USA

**Keywords:** Cell death, Neurophysiology

## Abstract

The non-conventional neurotrophic factor mesencephalic astrocyte-derived neurotrophic factor (MANF) is an endoplasmic reticulum (ER)-resident protein that promotes ER homeostasis. MANF has a cytoprotective function, shown in the central nervous system neurons and pancreatic beta cells. Here, we report that MANF is expressed in the hair cells and neurons and in selected non-sensory cells of the cochlea and that *Manf* inactivation triggers upregulation of the ER chaperones in these cells. However, *Manf* inactivation resulted in the death of only outer hair cells (OHCs), the cells responsible for sound amplification in the cochlea. All OHCs were formed in *Manf*-inactivated mice, but progressive OHC death started soon after the onset of hearing function. The robust OHC loss was accompanied by strongly elevated hearing thresholds. Conditional *Manf* inactivation demonstrated that MANF has a local function in the cochlea. Immunostainings revealed the upregulation of CHOP, the pro-apoptotic component of the unfolded protein response (UPR), in *Manf*-inactivated OHCs, linking the UPR to the loss of these cells. The phenotype of *Manf*-inactivated OHCs was distinctly dependent on the mouse strain, such that the strains characterized by early-onset age-related hearing loss (C57BL/6J and CD-1) were affected. These results suggest that *Manf* deficiency becomes detrimental when accompanied by gene mutations that predispose to hearing loss, by intensifying ER dyshomeostasis. Together, MANF is the first growth factor shown to antagonize ER stress-mediated OHC death. MANF might serve as a therapeutic candidate for protection against hearing loss induced by the ER-machinery-targeting stressors.

## Introduction

Mesencephalic astrocyte-derived neurotrophic factor (MANF) belongs to the large family of neurotrophic factors (NTFs), which regulate the life and death of both neuronal and non-neuronal cells. MANF was first characterized as a factor promoting the survival of midbrain dopamine neurons in vitro^1^. Later, the delivery of recombinant MANF protein to the rodent brain revealed neuroprotection against traumas^2,3^. Analysis of the *Manf* loss-of-function mutant mice has shown that the insulin-producing beta cells of the pancreas are particularly sensitive to *Manf* deficiency; they die from the juvenile stages onward, leading to diabetes^4^. The mouse strain has an impact on the *Manf* knock out (KO) phenotype: KO mice under the C57BL/6J (BL6) background die perinatally due to respiratory problems, whereas most KOs under the CD-1 background survive to adulthood^4,5^. Thus, available data give evidence that MANF promotes cell survival in a cell context-dependent and genetic background-dependent fashion.

MANF resides in the endoplasmic reticulum (ER) where it promotes protein-folding homeostasis^6^. MANF interacts physically with the major ER chaperone, the 78 kDa glucose-regulated protein (GRP78; also known as the binding immunoglobulin protein, BiP)^7,8^. Proteostasis defects trigger GRP78 and MANF upregulation, in conjunction with the activation of the unfolded protein response (UPR) and calcium release from the ER stores^9,10^. Besides promoting ER homeostasis and cell survival in a cell-autonomous fashion^6,11,12^, MANF has been suggested to function non-cell-autonomously as a secreted protein via still unclear extracellular mechanisms^7,13,14^.

Cochlear hair cells are mechanoreceptor cells that receive, amplify, and transduce acoustic stimuli. Inner hair cells (IHCs) are responsible for the sensory transmission to the brain, via the innervating spiral ganglion (SG) neurons. Outer hair cells (OHCs) amplify acoustic signals and are thereby critical for normal hearing sensitivity. OHCs are vulnerable to various stressors and their dysfunction or death is a common reason for sensorineural hearing loss. OHC vulnerability differs between mouse strains. For example, the BL6 and CD-1 mice display early-onset age-related hearing loss, as opposed to the CBA/Ca mice^15–18^. ER stress has been previously linked to hair cell loss and hearing loss^15,19,20^. There is no available data on the role of MANF in the inner ear. We have here focused on MANF’s expression in this tissue, on its impact on cell survival and on the underlying mechanisms. This knowledge is needed for understanding the role of ER homeostasis in auditory physiology and pathophysiology.

## Materials and methods

### Experimental animals

#### Manf KO mouse line

The original *Manf* KO mouse line was maintained under the CD-1 background (Envigo, RRID:MGI:5658486)^4^. In order to avoid early-onset hearing loss that characterizes the CD-1 strain^15,16^, we crossed *Manf* heterozygous mice with the CBA/Ca strain (Envigo, RRID:MGI:2159826), which maintains good hearing until old age^18^. We analyzed cochleas of *Manf* KOs and wildtypes of the F2 generation of CD-1 × CBA/Ca hybrids. *Manf* KO mice suffer from diabetes^4^ and due to poor health, they could not be maintained beyond 8 weeks of age. The following ages were used for cochlear analysis: postnatal day 2 (P2), P12, and 4 and 8 weeks. Mice of both sexes were included in the analysis. We used heterozygous mice of this *Manf* KO line for X-gal histochemistry to study *Manf* expression, because the *Manf* KO allele includes a *LacZ* gene construct. It was inserted between exons 2 and 3, with an efficient splicing acceptor ensuring β-galactosidase expression controlled by the *Manf* promoter^4^.

#### Manf conditional mouse line

To obtain *Manf*^*flox/flox*^*;Pax2-Cre* conditional KO (cKO) mice, we crossed the *Manf*^*flox/flox*^ and *Pax2-Cre* lines^4,21^, both under the BL6 background (C57BL/6JRccHsd, RRID:MGI:6151402, Envigo). Pax2 is expressed in the embryonic otic placode that gives rise to various epithelial cell types and neurons of the inner ear. It is also expressed in some other tissues, such as the developing midbrain–hindbrain boundary, and the developing and adult kidney^21^. We studied the cochlear phenotype of the cKO mice and littermate controls (*Manf*^*+/+*^*;Pax2-Cre* or *Manf*^*flox/+*^) before 11 weeks of age, because the BL6 mice are affected by early-onset hearing loss, the first signs of which can be seen at 3 months of age^17^. The following age groups, comprising both females and males, were studied: P2, P12, 5 weeks, and 8-to-11 weeks.

Blood samples were obtained from the tail vein and assayed for glucose concentration (MediSense Precision Xtra device, Abbott).

All animal work was conducted according to relevant national and international guidelines. Approval for animal experiments was obtained from the National Animal Experiment Board.

### Auditory brainstem response (ABR)

We anaesthetized mice via intraperitoneal injections of ketamine hydrochloride (75 mg/kg, Ketaminol vet, Intervet International Inc.) and medetomidine hydrochloride (1 mg/kg, Domitor vet, Orion Corporation). Mice were placed on a hot water bath to maintain body temperature at 37 °C, in an electrically and acoustically shielded chamber with echo damping padding. Subdermal needle electrodes were inserted at the vertex (active), below the right ear (reference), and below the pinna of the left ear (ground). MF1 Multi-field Magnetic Speaker was used to deliver monophasic click stimuli (0.1 ms duration, presented at a rate of 21/s) or 4–45 kHz tone-pip stimuli (1 ms duration, cosine-squared gated, presented at a rate of 21/s). The mouse was in a free-field setup with the speaker placed 10 cm from the right ear. Stimuli were presented from 90 to 10 dB sound pressure level (dB SPL) in 5 dB SPL steps. Electrical responses were obtained using the RA4LI headstage (20× gain amplification) directly connected to the RA4PA Medusa preamplifier/digitizer (250× amplification), which output to the RZ6 multi-I/O processor via fiber-optic cable. The responses were filtered (band-pass filter 300 Hz–3 kHz) and averaged (512 responses at each stimulus presentation level) using BioSigRZ software. The equipment was from Tucker-Davis Technologies (System 3). Thresholds were determined by visual inspection for the lowest sound intensity at which a repeatable waveform can be obtained. Calibration was performed with PCB-378C01 calibration microphone with model 480C02 sensor signal conditioner (PCB Piezotronics Inc.) using BioSigRZ and RPvdsEx virtual design studio software (version 84, Tucker-Davis Technologies).

### Cochlear whole mounts and immunostaining

We perilymphatically perfused cochleas with 4% paraformaldehyde (PFA), pH 7.4, in phosphate-buffered saline (PBS). This was followed by immersion in PFA for 2 h at room temperature or overnight at +4 °C. Cochleas were then decalcified with 0.5 M ethylenediaminetetraacetic acid (EDTA), pH 7.5, overnight at +4 °C. Organ of Corti and stria vascularis were dissected for whole mount specimens. Microdissection for the organ of Corti whole mounts has been previously described in Anttonen et al.^22^. Specimens were blocked with 10% goat serum (Jackson ImmunoResearch) in PBS containing 0.25% Triton-X-100 (PBS-T) for 1 h and then incubated with the primary antibody cocktail in PBS-T for 48 h at +4 °C. We used the following primary antibodies in double-labeling: rabbit polyclonal myosin7a (Myo7a; Proteus Biosciences Cat# 25-6790, RRID:AB_10015251, used at 1:3000), rabbit polyclonal MANF (Icosagen AS Cat# 310-100, RRID:AB_11135308, 1:500), rabbit monoclonal GRP78 (Abcam Cat# ab108615, RRID:AB_10890641, 1:1000), rabbit polyclonal calreticulin (Thermo Fisher Scientific Cat# PA3-900, RRID:AB_325990, 1:200), mouse monoclonal C-terminal-binding protein 2 (CtBP2; BD Biosciences Cat# 612044, RRID:AB_399431, 1:200), mouse monoclonal C*/EBP* homologous protein (CHOP; Novus Cat# NB600-1335SS, clone 9C3, RRID:AB_11033903, 1:100), and chicken polyclonal neurofilament heavy-chain (NF-H; Millipore Cat# AB5539, RRID:AB_11212161, 1:10,000). Primary antibodies were detected using AlexaFluor 488/594/647-conjugated goat anti-rabbit/mouse/chicken IgG or IgY secondary antibodies (Invitrogen). After antibody incubations, we visualized F-actin filaments using Oregon Green 514-conjugated or Rhodamine 568-conjugated phalloidin (Invitrogen). Nuclei were stained with DAPI. ProLong Gold anti-fade reagent was used for mounting (Invitrogen).

### Paraffin sections and histochemistry

We fixed and decalcified cochleas as above. We then embedded the specimens into paraffin (Histoplast IM, Thermo Fisher Scientific). We cut them in a midmodiolar plane into 5-µm-thick sections. Epitope retrieval was done by microwave heating (900 W) in 10 mM citrate buffer, pH 6.0, with a boiling time of 11 min. Sections were blocked with 10% goat or horse serum (Jackson ImmunoResearch) in PBS-T for 1 h and then incubated with primary antibodies for 48 h at +4 °C. We used the following primary antibodies: rabbit monoclonal GRP78 (Abcam, used at 1:10,000); rabbit monoclonal protein disulfide isomerase (PDI; Cell Signaling Technology Cat# 3501, RRID:AB_2156433, 1:1000), and goat polyclonal prestin (Santa Cruz Biotechnology Cat# sc-22694, RRID:AB_2190502, 1:1000). Detection was done with the Vectastain Elite ABC kit and diaminobenzidine substrate (DAB Detection kit). Sections were counterstained with methyl green. Some sections were used for hematoxylin–eosin staining (Hematoxylin and Eosin Stain Kit) (all from Vector Laboratories). Sections were mounted with Permount (Thermo Fisher Scientific). The intensity of immunostaining was compared between control and mutant sections that had been processed and imaged exactly in the same way.

#### Preadsorption assay

To confirm the lack of non-specific GRP78 staining, we incubated the primary antibody (1:6000 dilution; 0.16 µg/ml) with a 2.5-fold excess (0.4 µg/ml) of the immunizing peptide (Abcam) overnight at +4 °C. We then applied this mix on cochlear sections. The preadsorption abolished all staining in sections (See Results Fig. 4e).

#### Imaging

BX61 microscope equipped with UPlanApo ×10(NA 0.4) and ×40(NA 1.3, oil immersion) objectives was used for transmitted light images. They were taken with the DP73 CCD color camera and CellSens software (all from Olympus). Further image processing was performed in Adobe Photoshop CC 2017.

### X-gal staining

We dissected cochleas from heterozygote mice of the *Manf* KO line and perilymphatically perfused cochleas with 4% PFA, followed by immersion in the fixative at room temperature for 1.5 h. X-gal incubation was performed at +37 °C for 15 h. Cochleas were thereafter postfixed, decalcified, and processed for whole mounts or paraffin sections as earlier described in Herranen et al.^23^. In paraffin-embedding, deparaffination and dehydration of sections, the time in each alcohol step was kept to a minimum to avoid fading of X-gal staining. Sections were counterstained with 0.1% Nuclear Fast Red and mounted in Permount. Specimens were imaged with the BX61 microscope setup described above.

### Cochlear mapping and hair cell and synaptic ribbon counts

#### Total and regional OHC loss counts

We prepared a frequency map from each cochlea using the Measure Line.class ImageJ plugin (available from Eaton-Peabody Laboratories Histology Resources) and the calculations were done according to Müller et al.^24^. OHC loss was counted from DAPI/phalloidin/Myo7a/CtBP2-stained whole mounts. Lack of both the nucleus and the positively stained cell body was used as the criterion for the absence of a cell. OHC loss was counted across the cochlear duct, from the basalmost hook region to the apex. Image stack acquisition for cell counts was performed with Axio Imager.M2 microscope equipped with Apotome 2 structured illumination slider, using PlanApo ×10 objective (NA 0.45) and a *z*-step-size of 1.5 µm, CMOS camera (Hamamatsu ORCA Flash 4.0 V2) and Zen 2 software (all from Zeiss). Percentage of lost OHCs was counted from the total OHC population. In addition, 8-to-10-week old control and cKO mice were used to count the percentage of OHC loss at the 16, 32, and 45 kHz frequency regions. The frequency-specific cell counts were done from ×40 image stacks where the countable area included ~140 OHCs.

#### Presynaptic ribbon counts

Synaptic ribbons in the basal pole of IHCs were counted from the same whole mounts as used for OHC loss quantification, using the CtBP2 antibody to mark the synaptic ribbons. Ribbon quantification per IHC was performed at 16, 32, and 45 kHz cochlear regions from the maximum intensity projection images, which were processed with ZEN 2 software. *z*-stacks were obtained with Axio Imager.M2 microscope equipped with Apotome 2 structured illumination slider, using PlanApo ×40 objective (NA 1.3, oil immersion) and a *z*-step-size of 0.25 µm. The Hamamatsu ORCA Flash 4.0 V2 camera was used for image acquisition. Each image stack spanned from the cuticular plate to the synaptic pole of IHCs and contained the entire synaptic pole of ~40 IHCs.

### Statistical analysis

For all statistical analyses Origin 2018b (OriginLab Corporation) was used. Significant differences were reported with 95% confidence intervals. Error bars represent standard deviation (SD) except in ABR measurements where standard error of mean (SEM) was used. Welch’s *t*-test was used to compare the means of two experimental groups. One-way or two-way ANOVA with Tukey’s test was utilized for means comparison of multiple groups.

For the *Manf* KO line, KO and wildtype males were used for the comparison of the body weight and blood glucose concentration. In case of the cKO line, females were used for these assays. The body weight and glucose concentration were compared with Welch’s *t*-test (cKO) and one-way ANOVA (KO).

Two-way ANOVA was utilized for comparing the mean of ABR thresholds, OHC loss and synaptic ribbon numbers per IHC. ABR thresholds were compared between control and *Manf* KO or cKO mice at 4/5 weeks and at 8/8–11 weeks of age, respectively, in response to click and tone-pip stimuli. In the *Manf* KO line under CD-1 × CBA/Ca background, ABR thresholds and OHC loss of the *s*- and *l*-groups of KOs (see the section “Results”) were compared to wildtypes. Synaptic ribbon counts per IHC were compared between control and *Manf* cKO mice at three cochlear frequency regions.

## Results

### MANF is expressed in distinct cell types of the cochlea

**Fig. 1:**
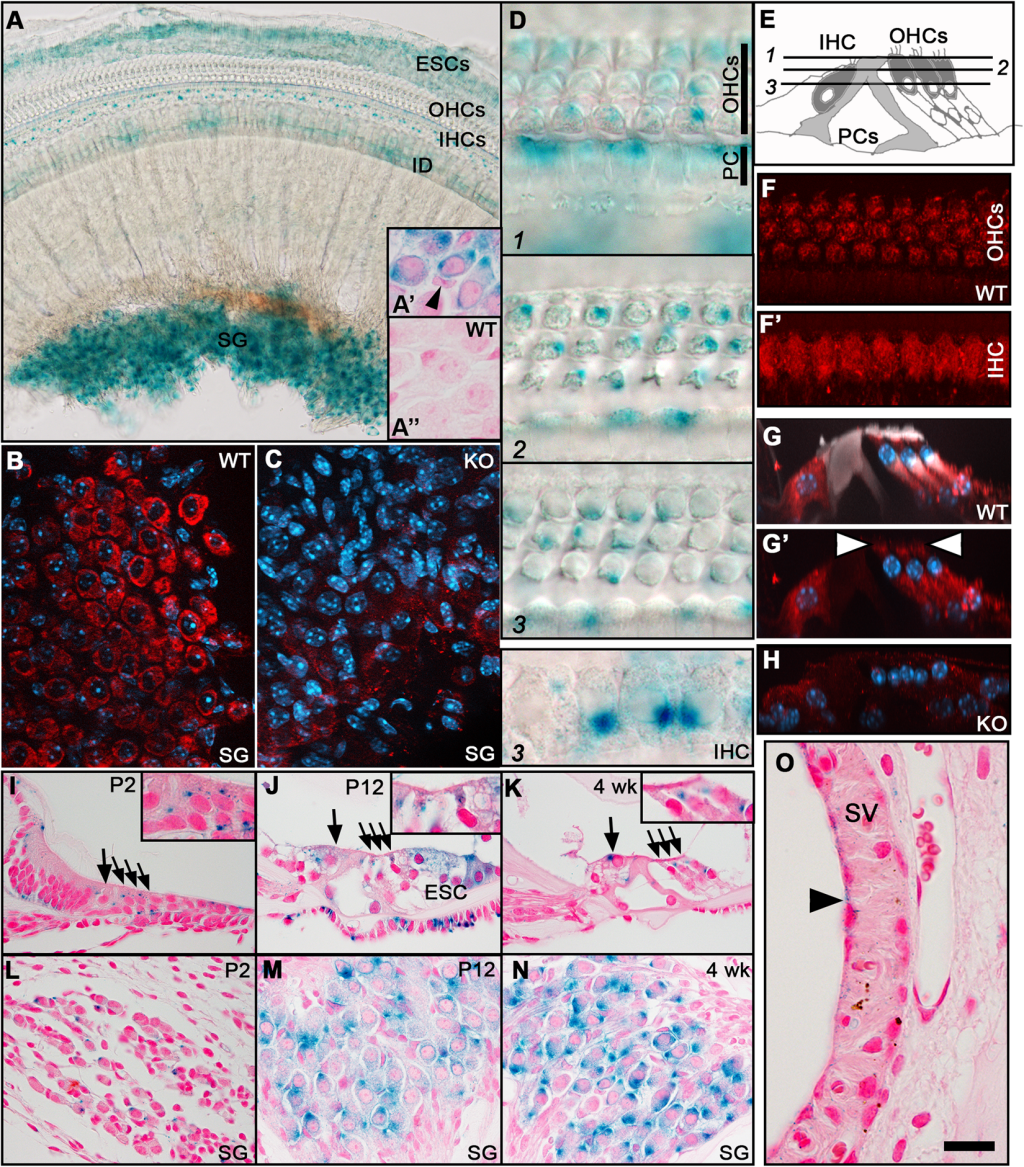
MANF expression in the cochlea, revealed by X-gal histochemistry and immunostaining. **a** The whole mount shows prominent X-gal staining in the SG, IDs, IHCs, and ESCs. The weaker staining in OHCs is not evident in this low magnification. **a**′ X-gal staining is present in SG neurons and absent in the satellite cells (arrowhead), revealed in a paraffin section. **a**″ X-gal staining is absent from SG neurons from a WT mouse. **b** MANF immunostaining (red) is prominent in SG neurons, revealed in a whole mount. DAPI marks nuclei (blue). **c** SG neurons from a KO mouse lack MANF staining. **d** Whole mount is viewed at different planes from the hair cell cuticular plate down to nuclear level. X-gal staining is prominent in IHCs and pillar cells (supporting cells). In OHCs, X-gal staining is concentrated to the plane below the cuticular plate (plane 2). Note, however, that X-gal staining is not a readout of intracellular protein distribution pattern. **e** Schematic cross-section through the organ of Corti depicts the imaging planes in **d** and the cell types. **f** Whole mount shows MANF immunostaining in OHCs, at the plane below the cuticular plate. **f**′ The same wholemount shows widespread MANF immunostaining in IHCs. **g**, **g**′ Maximum intensity projection of MANF-immunostained (red) whole mount viewed in transverse plane. Phalloidin (white) labels F-actin and DAPI (blue) nuclei. MANF is expressed below the cuticular plate (arrowheads) in OHCs. MANF is widely expressed in the IHC cytoplasm as well as in Deiters’ cells (supporting cells) beneath OHCs. Phalloidin labels the hair cell apices and pillar cells. **h** MANF immunostaining is absent from the organ of Corti of KO mice. **i**, **j**, **k** Transverse paraffin sections show X-gal staining in the organ of Corti at different ages. Large arrows mark IHCs, smaller arrows the three OHC rows. Insets show OHCs in higher magnification. **l**, **m**, **n** Transverse paraffin sections show the increase of X-gal-staining intensity along aging. **o** *Manf* is very weakly expressed in the luminally located marginal cells (arrowhead) of the stria vascularis. *Abbreviations*: KO knock out; WT wildtype; SG spiral ganglion; OHCs outer hair cells; IHCs inner hair cells; IDs interdental cells; ESCs external sulcus cells, PCs pillar cells, DCs Deiters’ cells; SV stria vascularis. Scale bar (in **o**): **a** 60 µm; **b**, **c** 20 µm; **d** 10 µm; **f**, **f**′, **g**, **g**′, **h**, **o** 15 µm; **i**–**n** 30 µm.

We used X-gal histochemistry and immunostaining to study MANF expression in the cochlea (Fig. 1a–o). We benefited from the design of the *Manf* KO allele^4^, including a *LacZ* gene construct that we detected by X-gal staining in heterozygote mice from the *Manf* KO line. X-gal staining was seen in the adult SG neurons and hair cells, IHCs showing stronger staining than OHCs. Epithelial cells lateral to the organ of Corti and interdental cells on the modiolar side were also stained (Fig. 1a, a′, d). This staining pattern was visible throughout the length of the spiraling cochlea. We confirmed the absence of non-specific X-gal staining in wildtype cochleas (Fig. 1a″).

To study *Manf* expression at younger ages, heterozygote cochleas were analyzed at P2, P12, and at 4 weeks of age. X-gal staining was relatively weak in the neonatal cochlea, but became stronger by P12, the stage representing the onset of hearing function. The staining intensity was maintained at this level at later ages (Fig. 1i–n). Marginal cells of the stria vascularis showed very weak staining at all ages studied (Fig. 1o; data not shown).

We performed MANF immunohistochemistry on cochlear whole mounts from wildtype mice. MANF was strongly expressed in the adult SG neurons and IHCs, and weaker in OHCs (Fig. 1b, f–h), in accordance with X-gal staining. MANF staining was detected throughout the IHC cytoplasm, whereas it was concentrated to the region just below the cuticular plate in OHCs (Fig. 1f, g′). We did not detect immunostaining in *Manf* KO cochleas, confirming the specificity of the antibody used (Fig. 1c, h). These findings are in line with prior data that MANF is an ER-resident protein^6^ and that ER is accumulated to the pericuticular region in OHCs^25^.

### Manf KO mice show progressive outer hair cell loss and elevated hearing thresholds

The original *Manf* KO mouse line was maintained under the CD-1 background^4^. CD-1 mice suffer from early-onset hair cell loss and hearing loss^15,16^. To reduce this genetic background effect, we crossed the *Manf* KO line with the CBA/Ca strain and analyzed F2 hybrids. We found that wildtype hybrids have a complete OHC population and their ABR thresholds are within the normal range, making the comparison to KO littermates feasible.

**Fig. 2:**
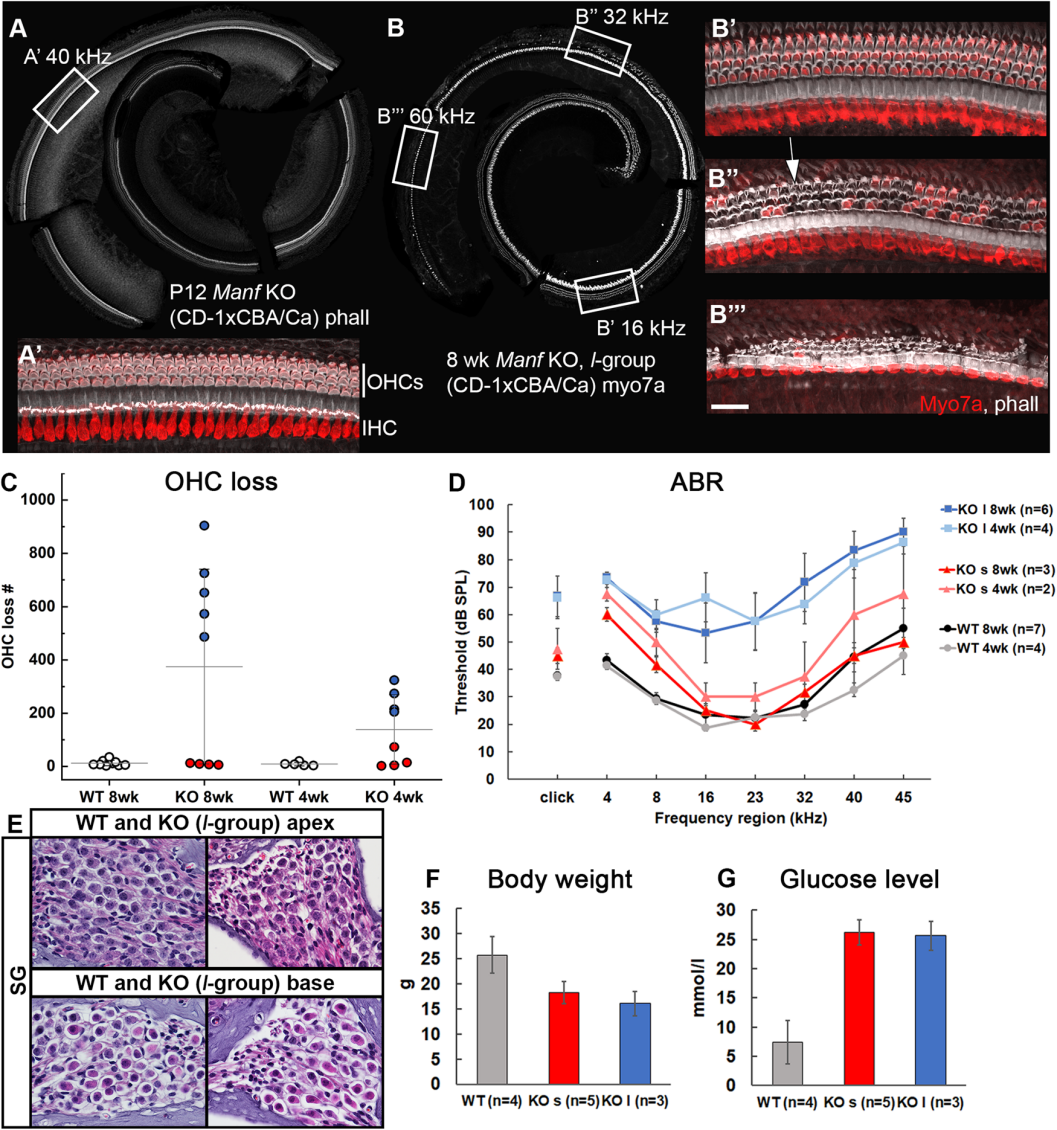
*Manf* KO mice under the CD-1 × CBA/Ca hybrid background show progressive OHC loss in a background-dependent fashion. **a**, **a**′ All hair cells are present in the KO cochlear whole mount at P12, revealed by phalloidin labeling. Boxed area in **a** marks the 40 kHz region, shown in a higher magnification in **a**′, with phalloidin (white) and myo7a (red) labeling. Phalloidin marks the apex of hair cells as well as pillar cells located between IHCs and OHCs. Myo7a marks the hair cell cytoplasm. **b** At 8 weeks of age, *l*-group of KO mice shows robust OHC loss from the 60 to 20 kHz region in the tonotopic axis, revealed by myo7a-staining (white). Boxed areas in **b** mark the frequency regions shown in higher magnifications in **b**′–**b**″′, with phalloidin (white) and myo7a (red) labeling. **b**′ All OHCs are present in the 16 kHz region. **b**″ A large part of OHCs are lost in the 32 kHz region. They are replaced by phalloidin-labeled F-actin scars (arrow in **b**″′), formed by Deiters’ cells. **b**″′ Most OHCs are lost in the 60 kHz region. Note that IHCs are present in this otherwise damaged organ of Corti. **c** Scatter plot with mean ± SD of OHC loss in KO and WT cochleas. KO cochleas are split into the *s*- and *l*-groups (red and blue dots, respectively), based on the extent of OHC loss. Each dot represents one mouse. **d** At 4 and 8 weeks of age, the difference in ABR thresholds between the KO *l*-group mice and WT littermates is statistically significant at all frequencies tested (two-way ANOVA, Tukey’s test, *P* < 0.05). This is also the case in the response to click stimuli (*P* < 0.05). ABR thresholds are comparable between the KO *s*-group and WTs at both age groups and at all frequencies tested, except at 4 kHz (two-way ANOVA, Tukey’s test, *P* < 0.01). Error bars represent SEM. **e** Hematoxylin–eosin-stained paraffin sections show a normal complement of SG neurons in the apex and base of the cochlea from the KO *l*-group. **f**, **g** The decrease in body weight and increase in blood glucose concentration are statistically significant between KOs and WT littermates. Males were used for analysis. Note that this difference applies to both the *l*- and *s*-group of KOs (one-way ANOVA, Tukey’s test, *P* < 0.001 for all comparisons). *Abbreviations:* KO knock out, WT wildtype; phall phalloidin; myo7a myosin7a; IHC inner hair cell, OHCs outer hair cells; ABR auditory brainstem response; SG spiral ganglion. Scale bar (in **b**″′): **a**, **b** 200 µm; **a**′, **b**′**–b**″′ 25 µm; **e** 35 µm.

We analyzed cochleas of *Man*f KO mice and wildtype littermates under the hybrid background at P12 and at 4 and 8 weeks of age. Cytocochleograms from KOs at P12 showed the presence of all hair cells (Fig. 2a, a′). However, a part of KOs displayed prominent OHC loss by 4 weeks and this loss progressed further, evidenced by a doubling by 8 weeks. OHC loss was concentrated to the basal part of the cochlea at 4 weeks and had extended to the middle part by 8 weeks (Fig. 2b, c). In line with the robust OHC loss, ABR thresholds were prominently elevated (Fig. 2d). The survival of IHCs and SG neurons was unaffected (Fig. 2b″, b″′, e). Surprisingly, in addition to the KOs with strongly affected OHC survival and ABRs, we found KO individuals with practically no change in these auditory parameters and, thus, they were comparable to wildtypes (Fig. 2c, d). Thereby, *Manf* KO mice formed two non-overlapping groups, the *large* and *small* group (*l-* and *s*-group based on the extent of OHC loss and ABR rise (Fig. 2c, d). At 8 weeks, on average 668 ± 159 OHCs (*n* = 5 mice) were lost in the *l*-group, while only 9 ± 3 OHCs (*n* = 4 mice) were absent in the *s*-group (Fig. 2c). Compared to wildtypes, the difference in OHC numbers was statistically significant in the *l*-group, both at 4 and 8 weeks of age (two-way ANOVA, Tukey’s test, *P* < 0.05 at 4 weeks and *P* < 0.001 at 8 weeks). Correspondingly, ABR thresholds were significantly elevated only in the *l-*group (Fig. 2d). Of note, both *l*- and *s*-group mutants showed other phenotypic changes typical to the *Manf* KO mice, namely hyperglycemia and reduced growth (Fig. 2f, g)^4,5^.

These results point to genetic heterogeneity of the *Manf* KO mice under the hybrid (CD-1 × CBA/Ca) background. Support for this conclusion came from our analysis of *Manf* KO mice under the pure CD-1 strain, a strain characterized by early-onset hearing loss^15,16^. These KOs showed extensive OHC loss and, notably, all individuals showed this robust effect (578 ± 236, *n* = 8 mice, 8 weeks of age). Thus, under the hybrid background, *Manf* inactivation combined with the hearing loss predisposing CD-1 background seem to characterize the *l*-group, while *Manf* inactivation together with the CBA/Ca status characterize the *s*-group.

### Conditional inactivation suggests a local function for MANF in the cochlea

**Fig. 3:**
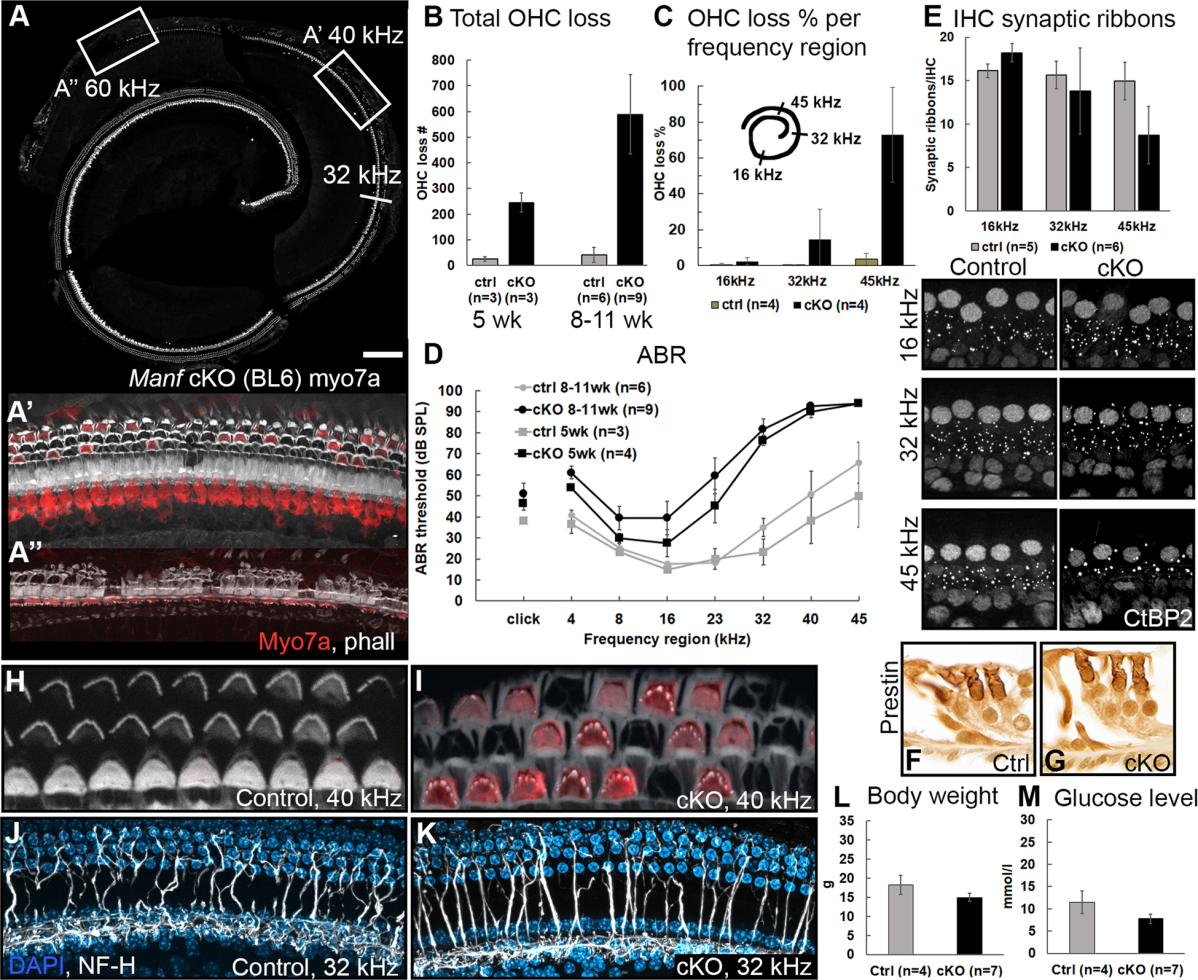
*Manf* cKO mice under the BL6 background show progressive OHC loss. **a** The whole mount from a 8-week-old *Manf*^*flox/flox*^*;Pax2-Cre* mouse shows extensive loss of myo7a-positive OHCs in the basal part of the cochlea. The loss continues up to the 20 kHz region. Boxed areas represent higher magnifications shown in **a**′ and **a**″. **a**′, **a**″ A subset of OHCs is preserved at 40 kHz **a**′, but OHC loss is near-total at 60 kHz **a**″, revealed by phalloidin (white) and myo7a (red) labeling. Note that also one IHC **a**″ and several pillar cells **a**′, **a**″ are lost in this strongly degenerated organ of Corti. **b** Mean OHC loss in cKO mice at 8-to-11 weeks of age is statistically significant compared to age-matched controls (two-way ANOVA, Tukey’s test, *P* < 0.001). Total OHC loss doubles in 5 weeks when comparing cKO mice at 5 and 8-to-11 weeks of age. Error bars represent SD. **c** The progressive OHC loss shown as the percentage of lost OHC at frequency regions 16, 32, and 45 kHz in 8-to-10-weeks old cKO and ctrl mice. Error bars represent SD. **d** In both age groups (5 weeks and 8-to-11 weeks), the rise in ABR thresholds in KO mice is statistically significant at high-frequencies, where also OHC loss is robust, compared to controls (two-way ANOVA, Tukey’s test, *P* < 0.05). Error bars represent SEM. **e** Synaptic ribbon counts (mean ± SD) in cKO mice (8-to-11 weeks of age) are comparable with age-matched controls at 16 and 32 kHz, but a statistically significant decrease in ribbon numbers is seen at 45 kHz (two-way ANOVA, Tukey’s test, *P* < 0.05). In the three frequency regions, CtBP2-stained synaptic ribbons were counted per IHC from maximum intensity projections of whole mount z-stacks. **f**, **g** Transverse paraffin sections show prestin expression in the OHC lateral wall of both genotypes. **h**, **i** Whole mounts from the basal part of cochleas of cKO and control mice were labeled with phalloidin (white) and myo7a antibody (red) and imaged at the level of the OHC stereociliary bundles. The V-shaped bundles of cKOs show a degenerative appearance compared to the cohesive bundles of controls. Due to the slightly different orientation of the specimens, cytoplasmic myo7a staining is not readily seen in the control specimen. **j**, **k** Maximum intensity projections of NF-H-stained whole mounts display afferent innervation (white) to OHCs that is comparable in cKO and control cochleas. DAPI (blue) marks nuclei. The images are from the middle part of the cochlea where OHC loss is scattered. **l**, **m** Body weight and blood glucose concentration are comparable between 8-to-11-week-old cKOs and control littermates. Females were used for this analysis (Welch’s *t*-test, *P* < 0.05). *Abbreviations*: cKO conditional knock out; myo7a myosin7a; phall phalloidin; ABR auditory brainstem response; OHC outer hair cell; ctrl control; CtBP2 C-terminal binding protein 2; NF-H neurofilament heavy-chain. Scale bar (in **a**): **a** 175 µm; **a**′, **a**″, **j**, **k** 20 µm; **e** 10 µm**; f**, **g** 25 µm; **h**, **i** 7 µm.

To rule out the effects of hyperglycemia on OHC viability and to obtain more understanding of the genetic background effect, we generated *Manf*^*flox/flox*^*;Pax2-Cre* cKO mice where *Manf* is inactivated in the epithelial cells and neurons of the inner ear^21^. This conditional inactivation approach does not target the beta cells of the pancreas and, correspondingly, we found comparable glucose levels in cKO and control littermates. Also the body weight was comparable between the genotypes (Fig. 3l, m).

We maintained *Manf* cKO mice under the pure BL6 background. As BL6 mice show initial signs of age-related hearing loss at 3 months of age^17^, we restricted our analysis to younger ages. Heterozygote *Manf*^*flox/+*^*;Pax2-Cre* cochleas did not show OHC loss (data not shown). cKO mutants showed a complete hair cell population at P12. Prominent OHC loss had developed in the basal part of the cochlea by 5 weeks of age and it progressed to the middle part, based on the analysis between 8 and 11 weeks of age (Fig. 3a–c). The cell loss accounts for ~25% of the total OHC population (about 2500 OHCs in the mouse cochlea). Consistent with this OHC loss, ABR thresholds were strongly elevated (Fig. 3d). Changes in both of these parameters were similar as in the *l-*group of *Manf* full-KO mice. Also similar to these full-KOs, the survival of IHCs and SG neurons was unaffected, except for a few IHCs that were absent in the region of strongest OHC loss (Fig. 3a–a″, data not shown). Together, MANF has a local function in the cochlea and OHCs are the major targets of *Manf* inactivation. Notably, as opposed to *Manf* KOs under the hybrid background, cKOs under the BL6 background showed a uniform cochlear phenotype.

Despite MANF expression in IHCs and SG neurons, *Manf* inactivation did not abrogate the survival of these cells. IHCs are connected to SG neurons by ribbon synapses, ~16 ribbons per IHC. We next asked whether the maintenance of these ribbon synapses is affected by *Manf* inactivation, taking into account that ER stress is linked with changes in calcium dynamics and that the ribbon synapse physiology depends on intracellular calcium buffering^26^. We prepared whole mounts of cKO and control cochleas between 8 and 11 weeks of age and quantified the CtBP2-positive presynaptic ribbons per IHC. cKOs showed decreased ribbon numbers in the basal portion of the cochlea, while the counts in the upper portion were comparable to controls (Fig. 3e). Thus, *Manf* inactivation triggered the loss of synapses between IHCs and SG neurons, and this synaptopathy was concentrated to the high-frequency region of the cochlea where also OHC loss was prominent. OHCs are also connected to SG neurons by ribbon synapses, with 2–4 ribbons per OHC. These CtBP2-positive ribbons were maintained in surviving OHCs of *Manf*-inactivated cochleas (data not shown).

We next focused on structural aspects of *Manf*-inactivated OHCs. The motor protein prestin underlies the active sound amplification by OHCs. Prestin is expressed in the OHC lateral wall. Immunostainings revealed that prestin is similarly expressed in OHCs of cKO and control cochleas (Fig. 3f, g). We used phalloidin labeling to reveal possible structural alterations in the OHC stereociliary bundles. At P12, no obvious differences were found between the genotypes (data not shown). However, adult cKOs displayed abnormal OHC bundles in the basal portion of the cochlea, showing up as absence or fusion of stereocilia (Fig. 3h, i). As OHC loss was robust in the same region, the bundle dysmorphology seems to be linked with the death-prone phenotype of *Manf*-inactivated OHCs.

We did not find evidence of perturbed SG neuron survival in *Manf*-inactivated cochleas (Fig. 1e). To rule out possible defects in the SG afferent innervation to OHCs, a defect that could promote OHC loss^27^, whole mounts of adult cochleas were stained with the NF-H antibody^28^. In the middle part of cKO cochleas where a many OHCs were still present, the afferent innervation pattern to OHCs was comparable to control specimens (Fig. 3j, k). Hence, we did not find a link between afferent innervation and abrogated OHC viability in *Manf*-inactivated cochleas.

### Manf inactivation causes upregulation of ER stress and UPR markers in the cochlear sensory cells

**Fig. 4:**
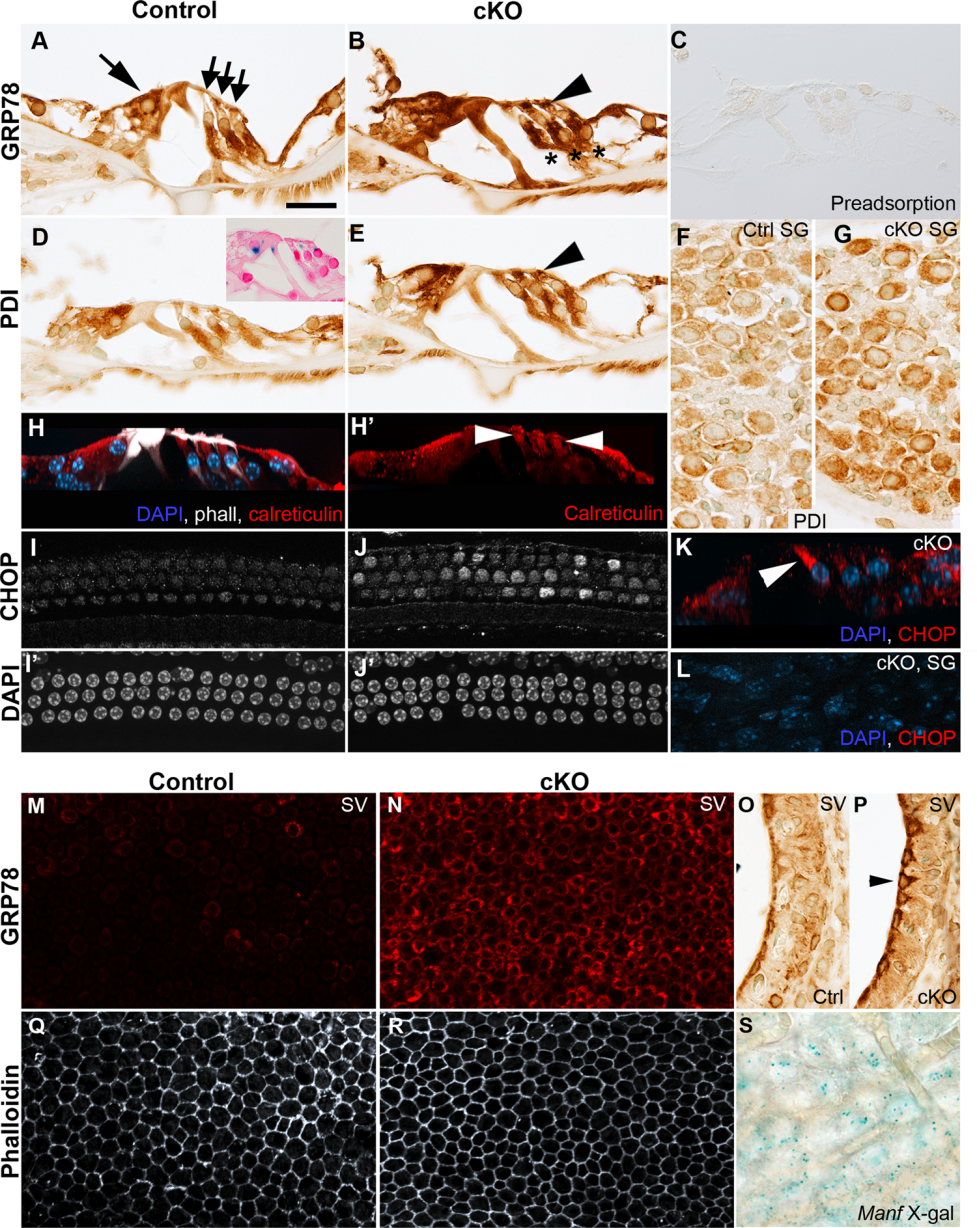
*Manf* inactivation triggers ER stress and UPR activation in the cochlea. Cochleas from 8-week-old *Manf*^*flox/flox*^*;Pax2-Cre* mice and control littermates were analyzed. Images were taken from the middle **a**–**l** or basal part of the cochlea **m**–**s**. **a**, **b** Transverse paraffin sections show ubiquitous GRP78 expression in the organ of Corti of control mice and GRP78 upregulation in cKOs. In OHCs, GRP78 is upregulated in the area below the cuticular plate (arrowhead). Large arrow marks IHC and small arrows OHCs. Deiters’ cells beneath OHCs are marked with stars. **c** GRP78 antibody was validated by preadsorption assay (see the section “Materials and methods”). **d**, **e** PDI shows also ubiquitous expression in controls and upregulation in cKOs. In OHCs, PDI upregulation has similar spatial characteristics (arrowhead) as GRP78. Inset in **d** is a reminder of *Manf* expression, shown by X-gal staining (see also Fig. 1). **f**, **g** Also SG neurons of cKO cochleas show PDI upregulation, revealed in paraffin sections. **h**, **h**′ The control whole mount is immunolabeled for calreticulin (red) and the maximum intensity projection is viewed in a transverse plane. Calreticulin expression pattern in OHCs (arrowheads) is comparable to GRP78 and PDI. Phalloidin (white) labels hair cell apices and pillar cells. DAPI labels nuclei (blue). **i**, **i**′, **j**, **j**′ Whole mount specimens show CHOP upregulation in OHCs of cKO cochleas. DAPI marks nuclei. **k** In the transverse section of a maximum intensity projection, CHOP expression (red) is seen in the first-row OHC. **l** Whole mount specimen from a cKO mouse shows absence of CHOP staining in SG neurons. DAPI marks nuclei (blue). **m**, **n** Stria vascularis whole mounts reveal GRP78 upregulation in marginal cells in cKO mice. **o**, **p** This upregulation in marginal cells (arrowhead) is also shown in paraffin sections. **q**, **r** Phalloidin labeling shows a regular hexagonal pattern of marginal cell boundaries, without signs of abrogated survival of these cells in cKO mice. **s** X-gal staining in a stria vascularis whole mount, viewed at the level of marginal cells, shows *Manf* expression (see also Fig. 1). *Abbreviations:* cKO conditional knock out; ctrl control; IHC inner hair cell; OHCs outer hair cells; ER endoplasmic reticulum; UPR unfolded protein response; SG spiral ganglion; GRP78 glucose-regulated protein 78; PDI protein disulfide isomerase; CHOP C/EBP homologous protein; SV stria vascularis. Scale bar (in **a**): **a**–**e**, **h**–**j**′, **l** 20 µm; **f**, **g** 25 µm; **k** 15 µm; **m**, **n**, **q**, **r** 37 µm; **o**, **p** 6 µm; **s** 12 µm.

MANF physically interacts with the ER chaperone GRP78 and this complex antagonizes ER stress caused by protein folding defects^7,8^. To find out if GRP78 is upregulated in *Manf*-inactivated cochleas, similar as shown in the pancreas^4^, we stained cochlear sections from 8-week-old mice with the GRP78 antibody. Wildtype cochleas showed widespread, moderate expression. cKO cochleas displayed GRP78 upregulation in the cells normally expressing MANF, including OHCs, IHCs, and SG neurons (Fig. 4a–c). PDI, another ER stress-induced chaperone, was upregulated in the same cells (Fig. 4d–g). In OHCs of cKO mice, these ER stress markers were concentrated to the pericuticular region, similar as the ER organelle marker calreticulin (Fig. 4h, h′). ER stress is associated with UPR activation. The pro-apoptotic UPR branch engages CHOP as a distal component. To find out if CHOP is induced in *Manf*-deficient OHCs, cochlear whole mounts from 8-week-old mice were stained with the CHOP antibody. As opposed to wildtype OHCs, CHOP was induced in a subset of OHCs in the basal part of cKO cochleas. This induction formed a front along the base-to-apex axis of the cochlea and it preceded the front of OHC loss (Fig. 4i–k). CHOP was not expressed in other cochlear cell types, consistent with the fact that cell death was confined to OHCs (Fig. 4l, data not shown).

### Manf deficiency does not abrogate the survival of marginal cells of the stria vascularis

Mechanotransduction by hair cells and the viability of these cells depend on the ionic composition of the endolymph. Marginal cells of the stria vascularis secrete ions into the endolymph. Strial whole mounts and sections from *Manf* cKO cochleas showed distinct GRP78 upregulation in marginal cells (Fig. 4m–p), consistent with *Manf* expression, albeit weak, in these cells in wildtype animals (Figs. 1o and 4s). However, phalloidin-labeled whole mounts showed a normal hexagonal pattern of marginal cell boundaries (Fig. 4q, r), indicating that *Manf* depletion does not disrupt the survival of these cells.

### Manf deficiency does not abrogate the survival of vestibular sensory cells

Finally, we studied whether the inner ear vestibular organs are affected by *Manf* inactivation. Hair cells of the utricle, saccule, and cristae as well as the vestibular ganglion neurons showed MANF expression, evidenced by X-gal staining and immunostaining (Supplementary Fig. 1a–e′, data not shown). However, vestibular organs of *Manf* cKO and full-KO mice did not show loss of Myo7a-positive hair cells and, consistently, these mutants lacked behavioral abnormalities associated with vestibular disorders, such as circling, head bobbing, and abnormal gait (Supplementary Fig. 1f, g, data not shown).

## Discussion

Here we have demonstrated by a genetic approach that MANF, a growth factor promoting ER homeostasis, is required for OHC survival. MANF was expressed in IHCs, OHCs, SG neurons, and in selected non-sensory cells of the cochlea. *Manf* inactivation triggered upregulation of the ER stress markers, the chaperones GRP78 and PDI, in these cells. However, it was primarily the OHC survival that was affected by *Manf* inactivation. Analogously, the pancreatic beta cells show a tight association between *Manf* inactivation, ER stress, and a death-prone phenotype^4^. We studied the inner ear phenotype of *Manf* KO mice. Because these mice suffer from diabetes, that might impair hearing function, we also generated non-diabetic *Manf* cKO mice. The mutants showed robust OHC loss and ABR threshold shifts. Thereby, we confirmed that MANF has a local role in the cochlea in promoting OHC survival and normal hearing function.

Local injection of the ER stress-activating drug tunicamycin into the rat cochlea has been shown to cause OHC death^19^. More recently, transmembrane and tetratricopeptide repeat 4 (Tmtc4), a broadly expressed regulator of ER calcium dynamics, was linked to ER stress and UPR activation in the cochlea^20^. It was shown that ER stress and UPR markers are induced in the cochleas of *Tmtc4* KO mice and that these mice suffer from hearing loss. These data support our results with the *Manf*-inactivated mice, showing that ER stress triggers OHC death and hearing loss. Cellular ER stress engages UPR activation that has an adaptive role aimed to restore proteostasis. Prolonged ER stress can activate the pro-apoptotic UPR branch with CHOP as a distal component^9^. We found CHOP induction in OHCs of *Manf*-inactivated cochleas, specifically in the area of ongoing OHC death. Thus, our results link the upregulation of ER chaperones and UPR signaling to OHC death. In wildtype OHCs, MANF had a distinct subcellular distribution, being expressed in the neck region beneath the cuticular plate. Prior studies have localized accumulation of ER and mitochondria to this region^25,29^. Correspondingly, we found GRP78 and PDI upregulation specifically in the neck region of *Manf*-deficient OHCs. Recently, we utilized serial block-face scanning electron microscopy to study trauma-induced pathology in the cochlear sensory epithelium^22^. In addition to that we localized mitochondria and cisternae to the OHC’s neck region, we found that this region becomes vacuolized in the late phases of degeneration. The OHC is then decapitated at this site, resulting in phagocytosis of the apoptotic cell fragments by neighboring supporting cells. These results indicate that OHC death is driven from the “reactive” neck region. Interestingly, IHCs do not show this kind of subcellular ER and mitochondria aggregation. Rather, these organelles are broadly distributed in the IHC cytoplasm^30^. This was also revealed in our MANF and GRP78 immunostainings. These differences might contribute to the differential vulnerability of IHCs and OHCs. IHCs are known to be more death-resistant than OHCs.

Not all OHCs were lost in *Manf*-inactivated cochleas; it was the high-frequency OHCs in the basal part of the cochlea that were the most sensitive. It is known that they possess differences in calcium-handling capacity compared to the low-frequency OHCs in the upper part of the cochlea^29^. In cells in general, ER machinery is a major regulator of intracellular calcium homeostasis, and ER stress is associated with elevated cytoplasmic calcium levels^10^. The high-frequency OHCs are most vulnerable to environmental stressors, such as noise and ototoxic drugs^29^. Thereby, there might be differences in the ER stress induction and in the UPR pathway function that dictate the calcium-dependent differential vulnerability of the high-frequency versus low-frequency OHCs.

The stria vascularis is responsible for the endolymph ion homeostasis that it is critical for OHC survival^31^. MANF was weakly expressed in the strial marginal cells, and GRP78 was upregulated in these cells of *Manf*-inactivated cochleas. However, the survival of marginal cells was unaffected. Although our results point to cell-intrinsic regulation of OHC death in *Manf*-inactivated cochleas, we cannot exclude the possibility that ER stress impairs the ion secretory function of marginal cells, possibly then potentiating the vulnerability of ER-stressed OHCs.

In the *Manf* KO mouse line under the hybrid background (CD-1 × CBA/Ca), KO individuals formed two non-overlapping groups when OHC loss and ABR thresholds were analyzed. These auditory parameters were unaffected in one group, while they were strongly affected in the other group. The hybrid background appears to underlie these differences. Our results suggest that *Manf* inactivation alone does not trigger OHC loss. Rather, *Manf* deficiency becomes detrimental when accompanied by gene mutations that predispose to hearing loss. Several mouse strains show early-onset hearing loss, starting in the high-frequency region and gradually extending to the low-frequency region of the cochlea. In the BL6 mice, mutations in the *cadherin 23* (*Cdh23*) gene, coding for a stereociliary tip-link protein, are known to be a major cause of age-related OHC loss and hearing loss^32^. When a *Cdh23* mutation was induced in the zebrafish hair cells, it triggered ER stress and resulted in hair cell death^33^. A later study with the mouse cochlea supported these results by showing that *Cdh23* mutation triggers ER stress-mediated death of OHCs^34^. In our *Manf* cKO mouse line under the BL6 background, all mutants displayed prominent OHC loss and hearing loss at ages prior to the onset of age-related hearing loss that characterizes this strain. This is consistent with the idea that the *Cdh23* mutation predisposes to *Manf* inactivation-induced hearing loss, mechanistically by potentiating ER stress. Similar to the BL6 mice, the CD-1 mouse strain shows early-onset hearing loss, yet the underlying gene mutations are uncharacterized. In contrast, the CBA/Ca strain does not suffer from age-related hearing loss. Therefore, in the *Manf* KO line under the CD-1 x CBA/Ca hybrid background, the KO individuals with the altered auditory parameters seem to possess the CD-1 genetic background, which intensifies ER dyshomeostasis in OHCs. This seems not to be the case with the unaffected KOs under the CBA/Ca background.

Of the NTF family members, MANF (this study) and the neurotrophins neurotrophin-3 and brain-derived neurotrophic factor promote the survival of cochlear cells, yet the site of action and mechanism of action are different. Neurotrophins are secreted from hair cells and they critically support the survival of developing SG neurons, by binding to neuronal plasma membrane receptors^35^. MANF is also expressed in hair cells, but it does not have a major role on SG neuron survival, not at least during the time frame used in our study. Also, the afferent innervation to OHCs appeared normal in *Manf*-inactivated mice, suggesting that the primary defect is at the OHC level. Furthermore, we did not find any obvious role for MANF during morphogenesis of the inner ear sensory structures. We found that MANF regulates OHC survival after the onset of hearing function, by promoting ER homeostasis in a cell-intrinsic fashion. There is evidence that MANF is secreted from cells and that it acts non-cell-intrinsically via still unidentified plasma membrane receptors^7,13,14^. We did not find direct evidence for this role in the cochlea. However, a putative secreted role of MANF might be linked with synaptopathy that we observed in *Manf*-inactivated cochleas or with the supporting cells that might regulate OHC survival by their MANF secretion.

Our results have translational implications. Zebrafish studies have suggested that ER stress is a proximal cause of the death of hair cells carrying gene mutations linked to the Usher syndrome, such as the *Cdh23* mutation^33^. Usher syndrome is a leading hereditary deafness syndrome in humans^36^. Furthermore, ER stress and UPR activation have been suggested to mediate noise-induced hearing loss in mice^20^. Similarly as proposed in Parkinson’s disease and diabetes^37^, MANF therapy might antagonize ER stress and thereby it could reduce or delay OHC death triggered by stressors that target the ER machinery.

## Supplementary information

Supplementary material
