# Supplementary material for: Deficiency of the ER-stress-regulator MANF triggers progressive outer hair cell death and hearing loss
Source: Cell Death Dis. 2020 Feb 6;11(2):100. doi: 10.1038/s41419-020-2286-6 (PMC7005028; doi:10.1038/s41419-020-2286-6)
Supplement: Supplementary file 1 — Supplementary material [file 41419_2020_2286_MOESM1_ESM.pdf]

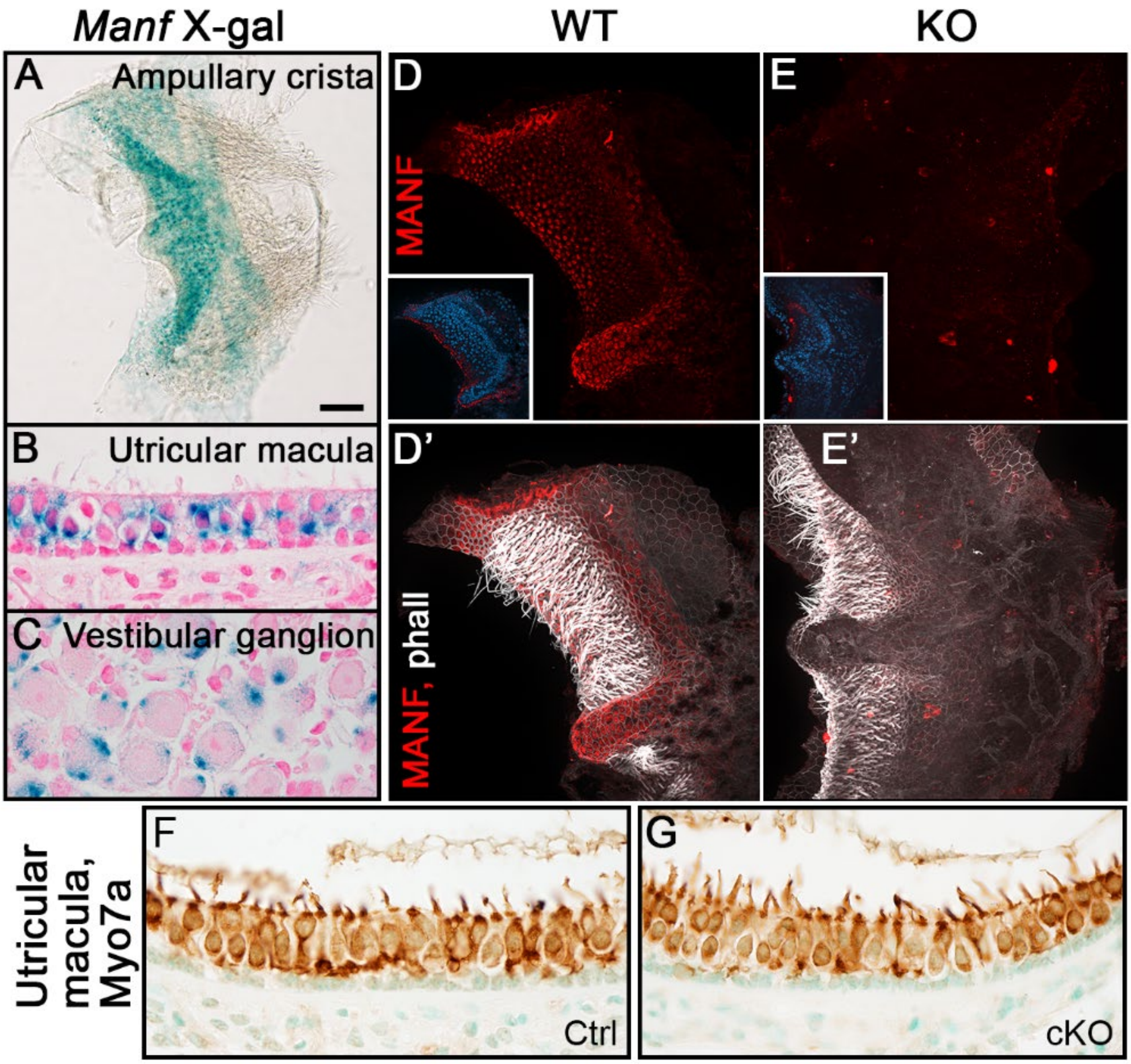

Supplementary Fig. 1. *Manf* inactivation does not abrogate the survival of vestibular hair cells. Vestibular organs were dissected from adult mice of the *Manf* KO or cKO line or from WT mice. **A**, Wholemount shows X-gal staining (see materials and methods) in the crista ampullaris. **B**, Paraffin section through the utricular macula shows X-gal staining in hair cells. Adjacent supporting cells are negative. **C**, Also vestibular ganglion neurons display X-gal staining, evidenced in a paraffin section. **D**,**D**′, MANF is expressed in hair cells of the crista ampullaris of WT mice, evidenced by co-labeling with phalloidin that strongly labels the stereociliary bundles. **E**,**E'**, MANF immunostaining is absent from the crista ampullaris of *Manf* KO mice. Insets in **D** and **E** display DAPI-labeled nuclei. **F**,**G**, Paraffin sections through the utricular macula show that there is no obvious loss of myo7a-positive hair cells in *Manf* cKO mice. *Abbreviations:* KO, knock out, cKO, conditional knock out; WT, wildtype; phall, phalloidin. Scale bar (in **A**): **A**, 70 µm; **B**,**C**,**F**,**G** 25 µm; **D-E'**, 40 µm.
